# Sedimentary evidence of invasive cladoceran *Bosmina (Eubosmina) coregoni* presence on the Pacific coast of North America over four decades before first detection

**DOI:** 10.1093/plankt/fbaf062

**Published:** 2025-11-28

**Authors:** Isaac Armstrong, Kathleen R Laird, Brian F Cumming

**Affiliations:** Paleoecological Environmental Assessment and Research Laboratory, Queen’s University, 116 Barrie St, Kingston, ON, K7L 3N6, Canada; Paleoecological Environmental Assessment and Research Laboratory, Queen’s University, 116 Barrie St, Kingston, ON, K7L 3N6, Canada; Paleoecological Environmental Assessment and Research Laboratory, Queen’s University, 116 Barrie St, Kingston, ON, K7L 3N6, Canada

**Keywords:** zooplankton, invasion ecology, Columbia river basin, lake sediments

## Abstract

While the invasion of Eurasian cladoceran *Bosmina (Eubosmina) coregoni* to the Laurentian Great Lakes is well-documented, its spread to the west coast of North America is less understood. We provide evidence that *Bosmina (Eubosmina) coregoni* was present on the Pacific Coast of North America as early as 1965, ~45 years prior to its first observation in this region in 2008. While investigating dated sediment cores in the Kootenay Lake system, British Columbia, Canada, we found *B. (E.) coregoni* remains in multiple intervals pre-2008, where it was present in high concentrations (up to 1500 exoskeletons/g dry sediment) and a dominant part of the cladoceran assemblage (25–31.5% relative abundance), though it displayed irregular pulses in abundance. While the method of introduction remains unclear, this research reframes the timeline of *B. (E.) coregoni* introduction and improves our understanding of zooplankton invasion dynamics.

## INTRODUCTION

The invasion of the Eurasian cladoceran *Bosmina (Eubosmina) coregoni* to the Laurentian Great Lakes is well-documented. *Bosmina (E.) coregoni* was first detected in Lake Michigan in 1968 following introduction by ballast water (Wells, 1970). It spread throughout the Great Lakes by 1971 and Manitoba by the 1980s (De Melo and Herbert, 1994b; Suchy *et al.,* 2010). The first Pacific Coast observation of *B. (E.) coregoni* is from 2008 in the Lower Columbia River Estuary (Smits *et al.,* 2013), though it is now established within the Columbia River basin (Dexter *et al.,* 2020). Due to the timing of this initial observation, it was unclear whether the invasion represented westward dispersion from the Great Lakes or a separate ballast water introduction from native populations in Asia (Dexter *et al.,* 2020). However, while conducting a paleolimnological investigation of the Kootenay Lake system, British Columbia, we found evidence that *B. (E.) coregoni* was present in the Pacific Coast of North America as early as the 1960s. Here we present concentration and relative abundance data for *B. (E.) coregoni* from four dated sediment cores and discuss possible methods of introduction and implications for detection of invasive cladocerans.

## METHODS

**Fig. 1:**
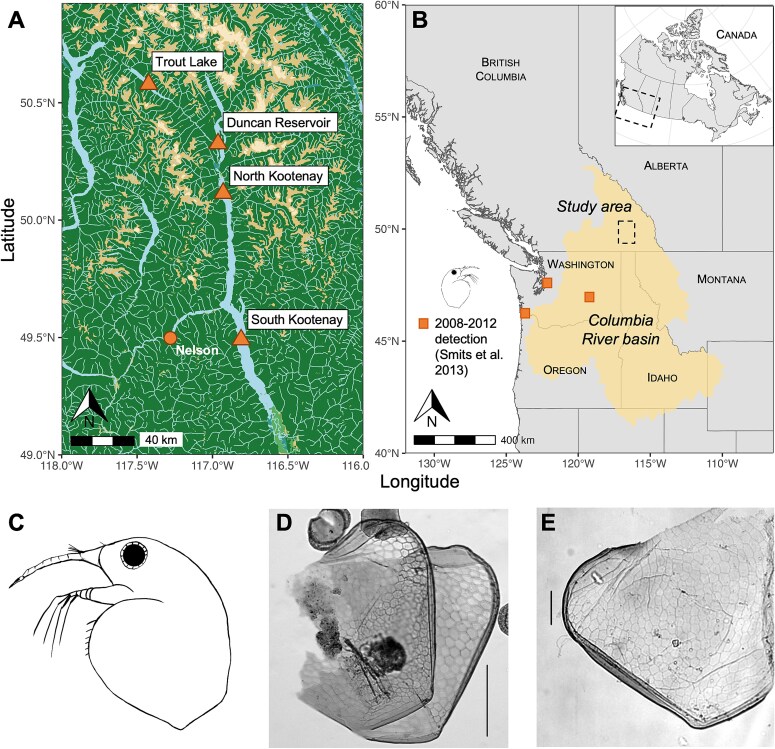
(A) Location of the four sediment cores. (B) Location of the study area within the Columbia River basin with approximate locations of *Bosmina (Eubosmina) coregoni* detection by Smits *et al.* (2013), with inset showing position relative to Canada. For spatial data sources please see ESM 1. (C) Line drawing of *Bosmina (Eubosmina) coregoni.* Illustration by author IA following De Melo and Herbert (1994a) (D) Photomicrograph of *Bosmina (Eubosmina) coregoni* carapace valves from the Kootenay Lake North Arm core, in sediments dated to ~1965. Scale bar 100 μm. (E) Photomicrograph of *Bosmina (Eubosmina) coregoni* carapace valves from a Great Lakes sediment core for comparison. Scale bar 50 μm (Panel Eadapted from Armstrong, 2024).

We examined cladoceran remains in sediment cores from three British Columbia lakes in the Upper Columbia River Basin: Kootenay Lake, Duncan Reservoir and Trout Lake (Fig. 1(A) and (B)). Sediment core chronologies were created with a Constant Rate of Supply model that tied the decay of unsupported ^210^Pb to ^137^Cs peak as an independent marker of 1963 (Figs S1 and S2) (Appleby, 2002). For post-1963 sediments, errors in all cores were relatively low (< ± 4 years). Sediment processing for cladoceran analysis followed established methods (Korhola and Rautio, 2001). While the exact temporal resolution of intervals varied between cores, Cladocera were generally analyzed at 0.5-cm (~1–2 year) intervals in post-1990 sediments and 1-cm intervals (~3–4 years) in pre-1990 sediments, though sediments prior to ~1930 could not be reliably dated. *Bosmina (Eubosmina) coregoni* carapace valves were identified by the lack of mucro (tail spine) and the strong reticulation (Fig. 1(C) and (D)) (Szeroczyńska and Sarmaja-Korjonen, 2007). *Bosmina (Eubosmina) coregoni* is the only known bosminid taxon in North America where the mucro is absent (De Melo and Herbert, 1994a), which allows for reliable identification. Further, the morphology and reticulation of carapace valves resembled *B.(E.) coregoni* subfossils identified in the Great Lakes (Fig. 1(E)), supporting our identification.

## RESULTS

**Fig. 2:**
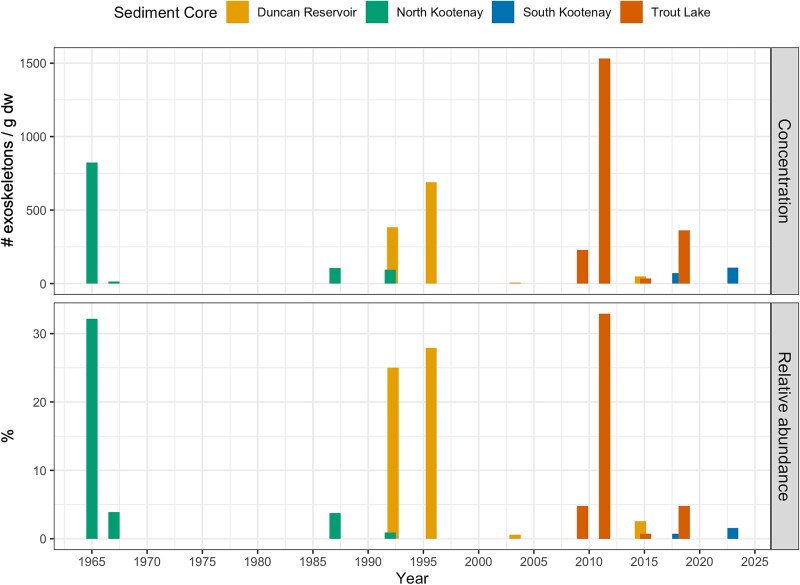
Detections of *Bosmina (Eubosmina) coregoni* in the four studied sediment cores by year according to the dating model. Top: concentration of remains (exoskeletons/g dry weight of sediment). Bottom: relative abundance (%) of *B. (E.) coregoni* out of all individual cladoceran exoskeletons counted for an interval.

High concentrations of *Bosmina (E.) coregoni* (822 exoskeletons/g dw) were found in sediment dated to 1965 in the North Arm of Kootenay Lake (Fig. 2), where it was 32% of the cladoceran assemblage. It was also present in 1967 (4%, 95 exos/g dw), 1987 (4%, 106 exos/g dw) and 1992 (1%; 95 exos/g dw), and remains were also found in two intervals pre-1930, though counted remains were too low (only 1–1.5 carapaces found) to reliably establish presence. In total, remains were found in six out of 44 intervals in the North Arm core. In the South Arm of Kootenay Lake, *B. (E.) coregoni* was found only in low abundances, present in two intervals (out of 43) which were dated to 2018 (0.7%, 109 exos/g dw) and 2023 (1.6%, 72 exos/g dw). In Trout Lake, *B. (E.) coregoni* was found in four out of 29 analyzed intervals, which were dated to 2009, 2011, 2015 and 2018. Its highest relative abundance was in 2011 (31.5%, 1 531 exos/g dw), while in the other intervals where it was present it was < 5% (34–360 exos/g dw). In Duncan Reservoir, it was present in three out of 33 intervals and was found in sediments dated to 1992 (25%, 382 exos/g dw), 1996 (27%, 690 exos g/dw) and 2016 (2.6%; 47 exos/g dw). Interestingly, *B. (E.) coregoni* remains were not found in the 1994 interval of Duncan Reservoir, despite its dominance in adjacent intervals. In all cores, intervals where *B. (E.) coregoni* had high relative abundances also included mucronate forms of *Bosmina* spp., which were generally the dominant cladoceran taxon present (40–60% relative abundance).

## DISCUSSION

*Bosmina (Eubosmina) coregoni* was evidently a substantial component of the cladoceran assemblage of Kootenay Lake as early as 1965. A likely vector of introduction is ballast water from commercial shipping between the Pacific Coast and estuaries in southeast Asia, which is noted as an invasion corridor for many zooplankton species (Dexter *et al.,* 2020). Our results suggests that this corridor was facilitating species introduction by the mid-1900s. Following its introduction, *B. (E.)* coregoni could have dispersed inland to Kootenay Lake through fish stocking, overland boat transport and/or gut passage in birds (Kelly *et al.,* 2013; Hessen *et al.,* 2019; Dexter *et al.,* 2020). Is it possible that *B. (E.) coregoni* could have rapidly dispersed westward from the Great Lakes? We believe it is unlikely, as the first observation of *B. (E.) coregoni* in the Great Lakes was in 1968 (Wells, 1970), though it may have been present prior to detection, and the timing of its westward spread to Lake Winnipeg (by the 1980s; Suchy *et al.,* 2010) suggests a slower rate of dispersal than would be required.

Interestingly, there is no record of *B. (E.) coregoni* from either historic (1949, 1964) or more modern (post-1990) zooplankton monitoring records (Zyblut, 1967; Schindler *et al.,* 2020) (see ESM for a note on systematics). This could be explained by the irregular pulses of abundance observed in the sediment cores. These unstable population dynamics are consistent with previous studies (Suchy and Hann, 2007; Smits *et al.,* 2013; Dexter *et al.,* 2015) and may represent repeated failed invasions. Alternatively, population blooms may be related to trophic state, as an increase in *B. (E.) coregoni* abundance is associated with eutrophication (Nilssen and Larsson, 1980) and cladoceran resting eggs can persist for decades in lake sediments when environmental conditions are unfavorable (Hairston Jr, 1996). In 1967, dam construction on the northern inflow of Kootenay Lake could have altered nutrient dynamics and favored *B. (E.) coregoni* over other bosminid taxa. However, for the other years in which *B. (E.) coregoni* was prevalent, we are unaware of any notable events that could have prompted population blooms. We are also unsure of what mechanism would delay the invasion of *B. (E.) coregoni* to upstream Trout Lake or why no *B. (E.) coregoni* pulses were detected in the sediment core from the South Arm of Kootenay Lake, though on local scales, invasive zooplankton distribution is primarily regulated by habitat structure, resources and biotic interactions, not by number of colonists or spatial proximity (Havel and Shurin, 2004; Havel *et al.,* 2015; Dexter *et al.,* 2020).

Due to the rapid morphological changes (cyclomorphosis) characteristic of bosminids, we must discuss whether the cladoceran we identify here as *B. (E.) coregoni* could be an unknown morphotype of an endemic species, such as *B. (E.) longispina*. However, *B. (E.) longispina* retain their mucros when undergoing cyclomorphosis (De Stasio Jr *et al.,* 1990), and co-existing populations of *B. (E.) coregoni* and *B. (E.) longispina* are usually distinct (Nilssen and Larsson, 1980). Speciation could have occurred from North American *Bosmina* population as has been described for other Holarctic *Bosmina* (*Eubosmina)* (Faustova *et al.,* 2011), but if so, we might expect taxa resembling *B. (E.) coregoni* to be more prevalent in North American lakes. Instead, its introduction in many eastern North American sediment records is congruent with Great Lakes invasion timing (Suchy and Hann, 2007; Suchy *et al.,* 2010; Armstrong, 2024). Thus, it is most likely our sediments are recording the invasion of Eurasian *B. (E.) coregoni*, though molecular analyses would be needed for confirmation (Kotov *et al.,* 2022).

In conclusion, our findings provide a new timeline for the invasion of *B. (E.) coregoni* to the Pacific Coast of North America. The irregular abundances shown by *B. (E.) coregoni* in our sediment cores is consistent with previous research and indicates a high temporal resolution is needed to accurately detect *B. (E.) coregoni* invasions.

## Supplementary Material

Supplementary_materials_fbaf062

## STATEMENTS AND DECLARATIONS

The initial study on the Kootenay Lake system where we observed this evidence of invasion was funded by a Natural Sciences and Engineering Research Council of Canada (NSERC) Alliance grant to BFC. The authors declare that no other funds, grants or support were received during the preparation of this manuscript. The authors declare no competing financial or non-financial interests.
