# Supplementary material for: Sedimentary evidence of invasive cladoceran Bosmina (Eubosmina) coregoni presence on the Pacific coast of North America over four decades before first detection
Source: J Plankton Res. 2025 Nov 28;47(6):fbaf062. doi: 10.1093/plankt/fbaf062 (PMC12661937; doi:10.1093/plankt/fbaf062)
Supplement: Supplementary_materials_fbaf062 [file supplementary_materials_fbaf062.docx]

**Supplementary Material for**

Armstrong, I., Laird, K.R., and Cumming, B.F. (2025) Sedimentary evidence of invasive cladoceran *Bosmina (Eubosmina)* *coregoni* presence on the Pacific Coast of North America over four decades before first detection. Submitted to Journal of Plankton Research

Corresponding author: Isaac Armstrong

[Isaac.armstrong@queensu.ca](mailto:Isaac.armstrong@queensu.ca)

**A note on systematics:**

*Bosmina* systematics are historically complex. The species *Bosmina (Eubosmina) longispina,* which possesses a mucro, has been previously referred to as *Bosmina coregoni*, *Bosmina coregoni longispina* (Patalas and Salki, 1973)*,* and *Eubosmina coregoni* (Wells, 1970; Brandlova *et al*., 1972), while the form without a mucro was specified as *Bosmina coregoni coregoni* (Nilssen and Larsson, 1980). Thus older monitoring records may document a species using the name *Bosmina coregoni*, but this is generally assumed to be referring to *B. (E.) longispina*, which is widespread in North America (De Melo and Herbert, 1994). For example, a Master’s thesis by Zyblut (1967) does record *Bosmina coregoni* in Kootenay Lake. However, Zyblut (1967) makes no distinction between mucronate and non-mucronate forms. As we found mucronate *Bosmina* spp. to be dominant in our sediment cores, and as it would have likely been remarkable to find a bosminid lacking a mucro in the core, we believe it likely that Zyblut (1967) was not referring to *B. (E.) coregoni*.

**
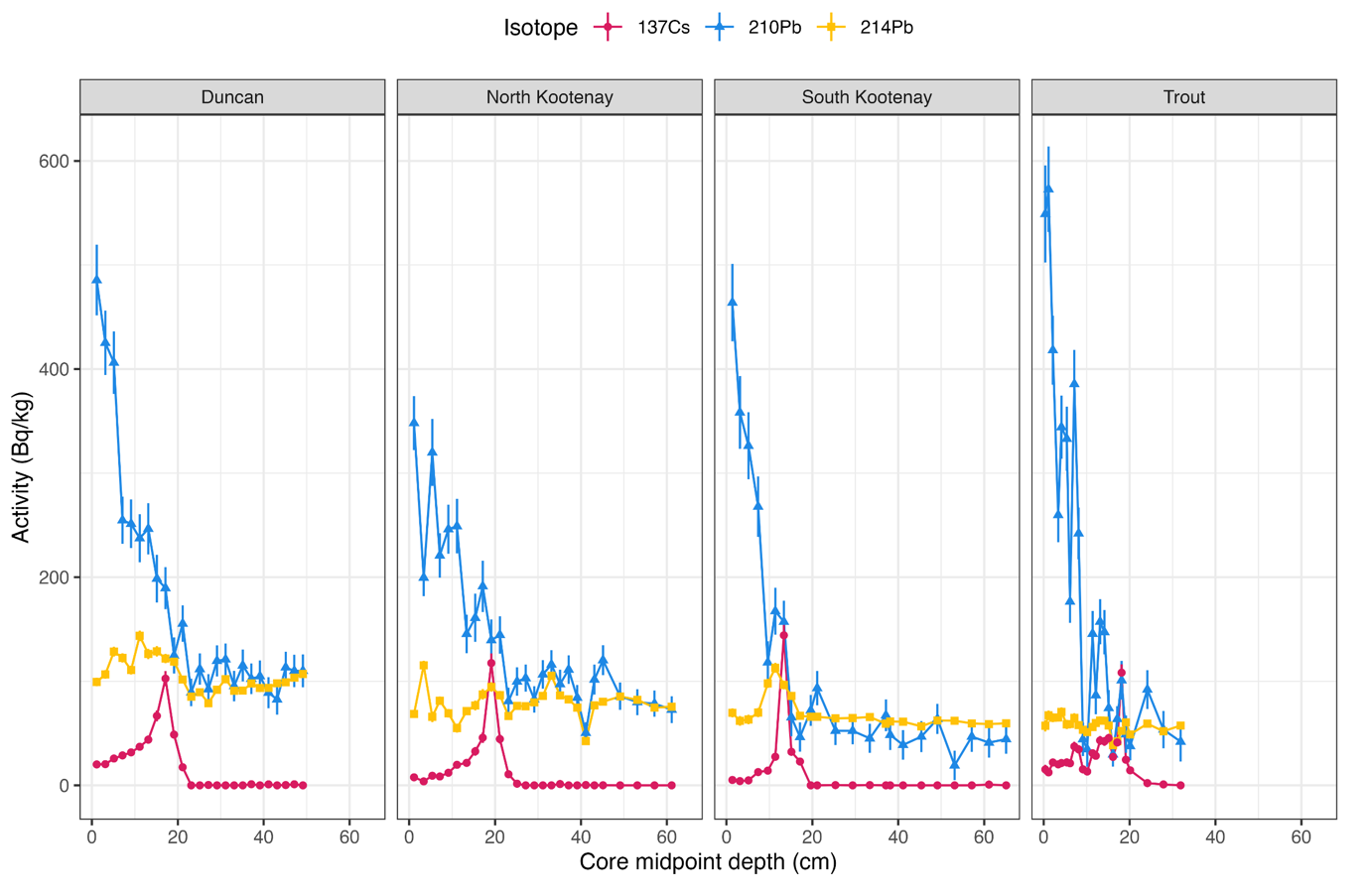
**

**Figure S1.** Activities (Bq/kg) of radioisotopes used to construct dating models of the four sediment cores. Specifically, the decay of unsupported ^210^Pb is measured by using ^214^Pb as a proxy for supported (background) ^210^Pb, and the ^137^Cs peak is used as an independent marker of 1963.

**
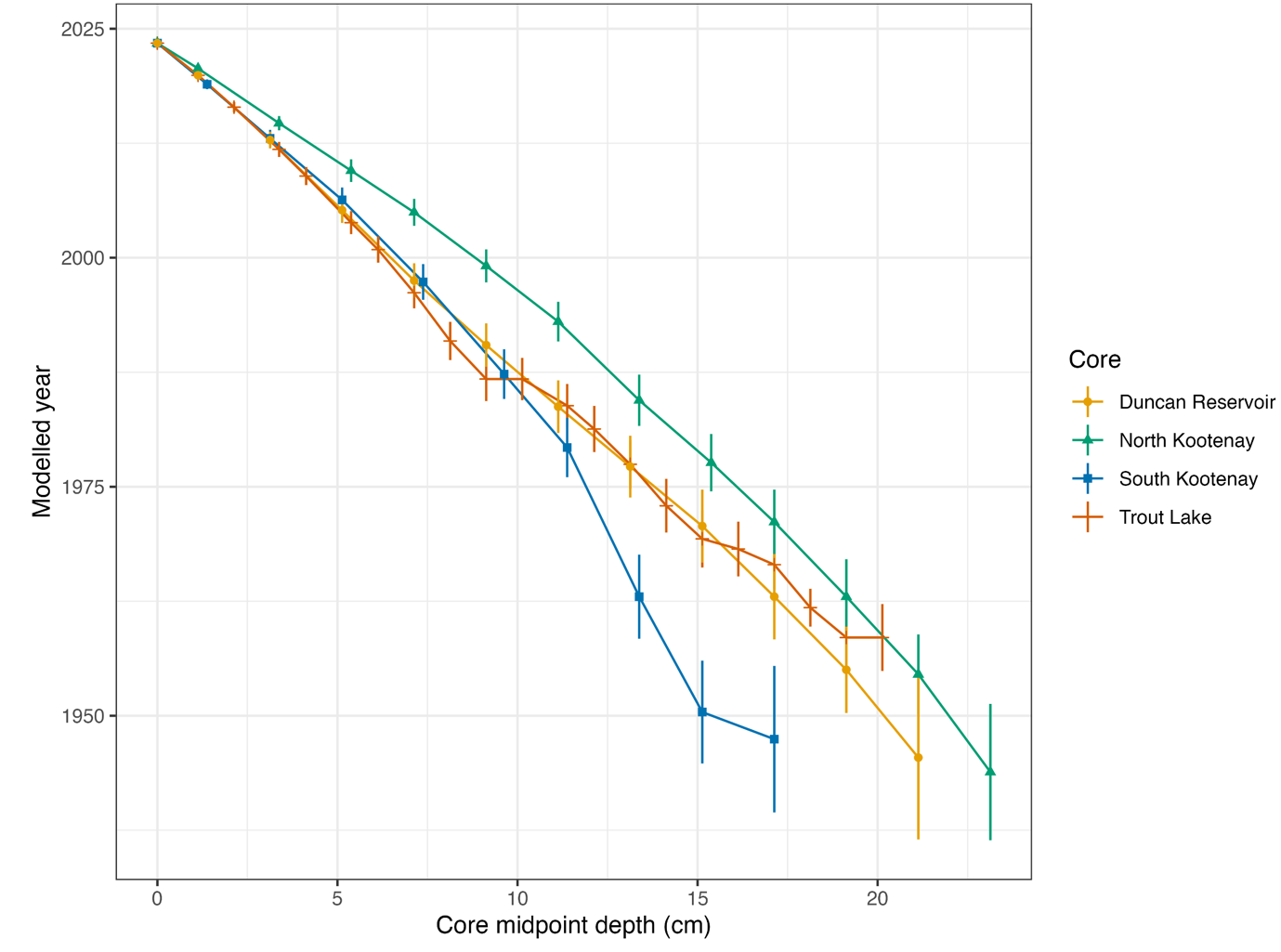
**

**Figure S2.** Sediment core dates and error margins given by midpoint depth (cm) for the four sediment cores. Dating models use a Constant Rate of Supply model tied to the ^137^Cs peak. Dating models are based on radioisotope activities shown in Figure S1, except for the South Kootenay core where a constant value of 50 was used instead of the ^214^Pb value due to anomalously high ^214^Pb activities.

**List of R packages**

Figures 1 and 3 were created using RStudio (R Core Team 2024; Posit team 2024) with general use of *dplyr* (Wickham et al. 2023) and *ggplot2* (Wickham 2016). Mapping was conducted with *sf* (Pebesma and Bivand 2023), *sp* (Bivand et al. 2013), and *ggspatial* (Dunnington 2023) with shapefiles from *canadianmaps* (Cayen 2024), *giscoR* (Hernangómez 2025), Natural Resources Canada (2017a,b,c), Pacific States Marine Fisheries Commission (2025), and the United States Census Bureau (2018).

**References**

Bivand, R., Pebesma, E., and Gomez-Rubio, V. (2013) Applied spatial data analysis with R, second edition. Springer, New York.

Brandlova, J., Brandl, Z., and Fernando, C.H. (1971) The Cladocera of Ontario with remarks on some species and distribution. *Can. J. Zool.,* **50**(11),1373-1403. https://doi.org/10.1139/z72-188

Cayen, J. (2024) canadianmaps: Effortlessly create stunning Canadian maps. R package version 2.0.0

De Melo, R. and Herbert, P.D.N. (1994) A taxonomic reevaluation of North American Bosminidae. *Can. J. Zool.*, **71**(10), 1808-1825. https://doi.org/10.1139/z94-245

Dunnington, D. (2023) ggspatial: Spatial data framework for ggplot2. R package version 1.1.9

Hernangómez, D. (2025) giscoR: Download map data from GISCO API – Eurostat. https://doi.org/10.32614/CRAN.package.giscoR

Natural Resources Canada (NRCAN). (2017a) Elevation in Canada – CanVec – Elevation Features (shapefiles).

Natural Resources Canada (NRCAN). (2017b) Lakes and rivers in Canada – CanVec – Hydro Features (shapefiles).

Natural Resources Canada (NRCAN). (2017c) Wooded areas, saturated soils and landscape in Canada – CanVec – Land features (shapefiles).

Nilssen, J.P. and Larsson, P. (1980) The systematical position of the most common fennoscandian *Bosmina (Eubosmina). J. Zool. Syst. Evol. Res.*, **18**, 62-68. https://doi.org/10.1111/j.1439-0469.1980.tb00727.x

Pacific States Marine Fisheries Commission (PSMFC). (2025) Columbia Basin Watershed Boundary (shapefile). Accessed from <https://hub.arcgis.com/datasets/PSMFC::columbia-basin-watershed-boundary/>

Patalas, K. and Salki, A. (1973) Crustacean plankton and the eutrophication of lakes in the Okanagan Valley, British Columbia. *J. Fish Res. Board Can.*, **30**, 519-542. https://doi.org/10.1139/f73-090

Pebesma, E. and Bivand, R. (2023) Spatial data science: with applications in R. First edition. Chapman and Hall/CRC, New York.

Posit team. (2024) RStudio: Integrated development environment for R. Posit Software, PBC, Boston, MA.

R Core Team. (2024) R: A language and environment for statistical computing. R Foundation for Statistical Computing, Vienna, Austria.

United States Census Bureau (USCB). (2018) Cartographic Boundary Files – Shapefile (cb_2018_us_state_5m). Accessed from https://www.census.gov/geographies/mapping-files/time-series/geo/carto-boundary-file.html

Wells, L. (1970) Effects of alewife predation on zooplankton populations in Lake Michigan. *Limnol. Oceanogr*., **13**, 556-565. https//doi.org/10.4319/lo.1970.15.4.0556.

Wickham, H. (2016) ggplot2: Elegant graphics for data analysis. Springer-Verlag, New York.

Wickham, H., François, R., Henry, L., Müller, K., and Vaughan, D. (2023) dplyr: A grammar of data manipulation*.* R package version 1.1.2

Zyblut, E.R. (1967) Temporal and spatial changes in distribution and abundance of macro-zooplankton in a large British Columbia lake (MSc thesis) University of British Columbia, Canada.
